# P-1966. Classification of Injection Drug Use by a Large Language Model Using Hospital Admission Notes

**DOI:** 10.1093/ofid/ofaf695.2133

**Published:** 2026-01-11

**Authors:** Edward C Traver, Seyed M Shams, Ishan Kumar Vaish, Jasmine Stevens, Meghan Derenoncourt, Hannah E Flores, Elana S Rosenthal, Sarah Kattakuzhy

**Affiliations:** University of Maryland School of Medicine, Baltimore, MD; University of Maryland - Institute for Health Computing, North Bethesda, MD; University of Maryland Medical School, Baltimore, Maryland; University of Maryland School of Medicine, Baltimore, MD; University of Maryland, Baltimore, Baltimore, Maryland; University of Maryland, Baltimore, Maryland; Institute for Human Virology (IHV), University of Maryland School of Medicine, Washington, District of Columbia; Institute for Human Virology (IHV), University of Maryland School of Medicine, Washington, District of Columbia

## Abstract

**Background:**

People who inject drugs (PWID) are at higher risk for severe bacterial infectious diseases (ID), which drive expensive hospitalizations. Identification of PWID allows for linkage to clinical interventions, such as multidisciplinary ID-addiction treatment teams, which improve clinical outcomes. Yet injection drug use (IDU) is often captured only in the text of clinical notes and is not easily queried. We sought to demonstrate text-based IDU classification by a large language model (LLM), a type of artificial intelligence.

**Figure 1:**
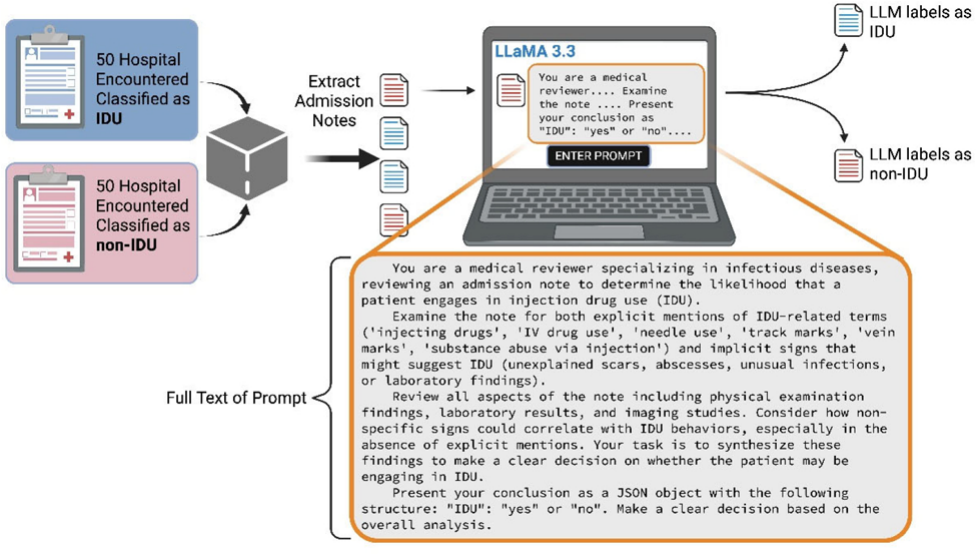
Workflow of the Classification and Labeling Procedures and Full Text of the Prompt. IDU, injection drug use; LLM, large language model; LLaMA 3.3 is the LLM used.

**Figure 2:**
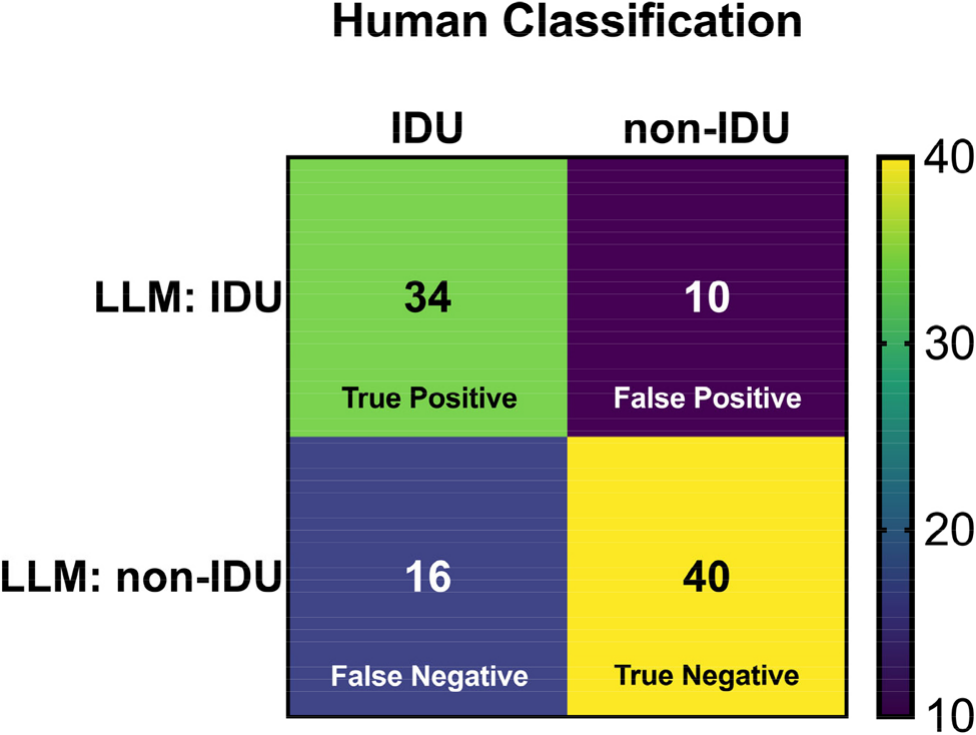
Confusion matrix of LLM labeling performance compared to human classification (treated as the ground truth). IDU, injection drug use; LLM, large language model.

**Methods:**

Hospital encounters at an academic medical center between 2018 and 2022 were included if they featured ICD codes for both acute infections and opioid use. Encounters were reviewed by trained research assistants and classified as “IDU” or “non-IDU” based on clinical notes. A balanced sample of 100 encounters was selected randomly for the LLM classification. The hospital admission note was extracted from the electronic medical record (Epic). A zero-shot prompt instructed the LLM (LLaMA 3.3; Meta, 70B parameters) to label each encounter as “IDU” or “non-IDU” (Figure 1). LLM labels were compared to human classifications. Positive and negative predictive values (PPV, NPV) were estimated for varying IDU prevalence. 95% confidence intervals were estimated with the Wilson-Brown method.

**Figure 3:**
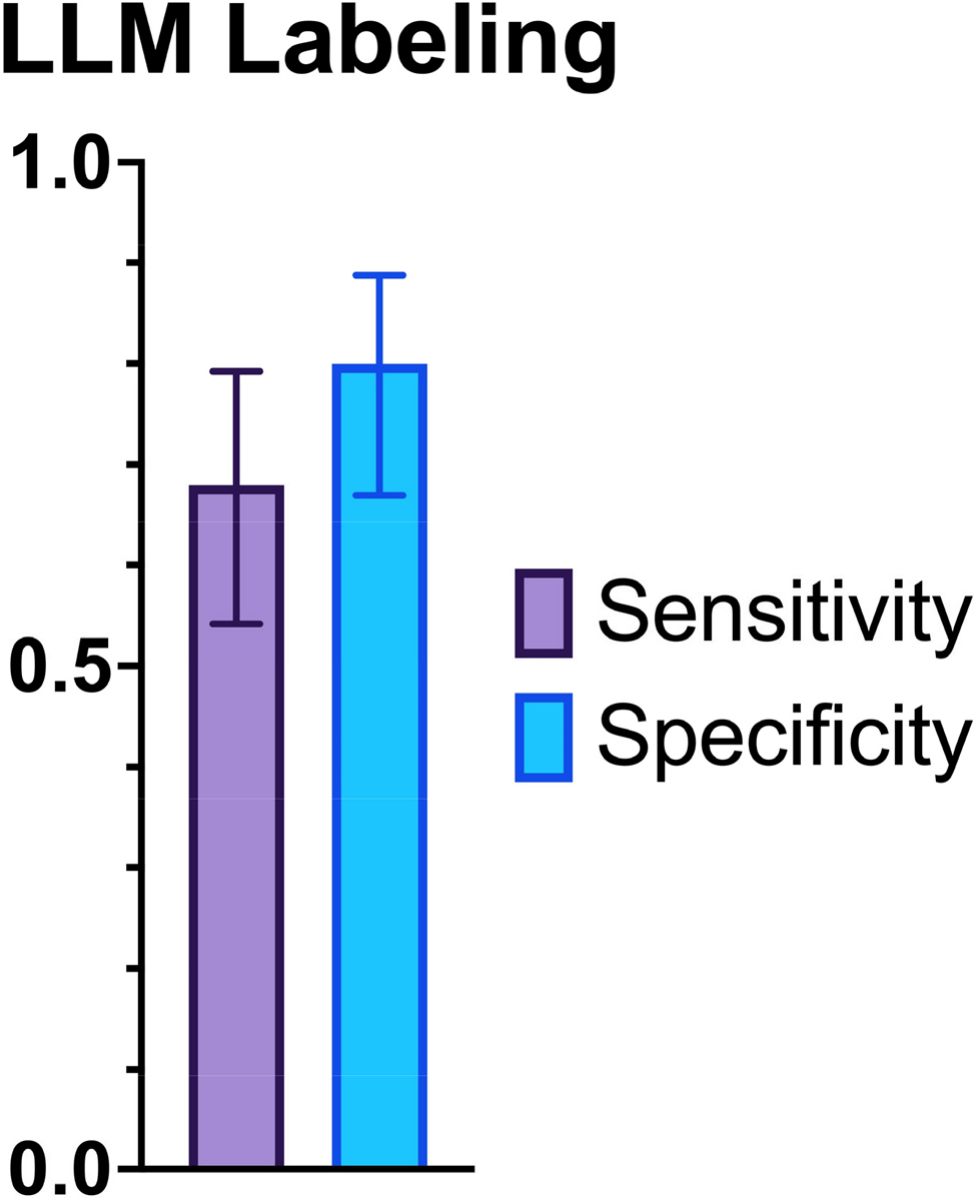
LLM labeling compared to human classification. Error bars, 95% CI

**Figure 4:**
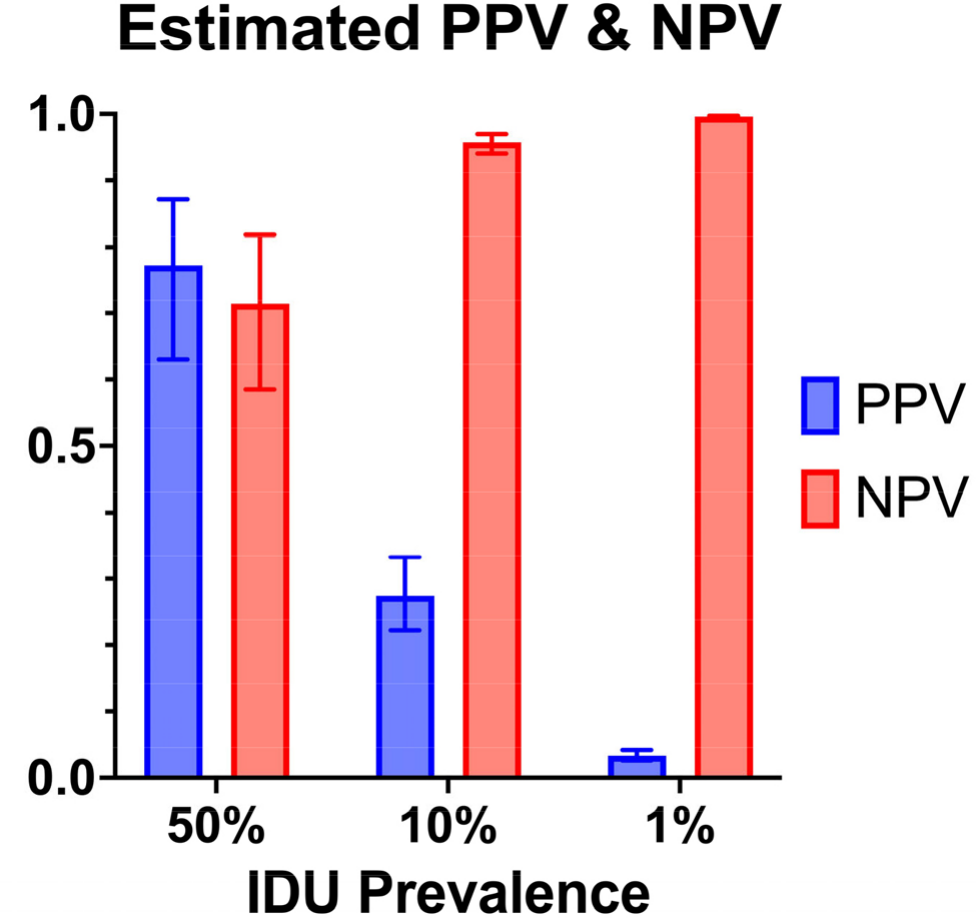
Estimated PPV and NPV of LLM labeling compared to classification in theoretical cohorts of varying IDU prevalence. IDU, injection drug use; LLM, large language model; NPV, negative predictive value; PPV, positive predictive value. Error bars, 95% CI.

**Results:**

Of the 50 IDU and 50 non-IDU encounters, the LLM labeling yielded 34 true positives, 16 false negatives, 40 true negatives, and 10 false positives (Figure 2). Sensitivity was 0.68 (95% CI 0.54-0.79); specificity 0.80 (95% CI 0.67-0.89; Figure 3). Accuracy of the LLM label was 0.74; F1-score 0.72. Estimates of PPV with IDU prevalence of 50%, 10%, and 1% were 0.77, 0.27, and 0.03; estimates of NPV were 0.71, 0.96, and >0.99 (Figure 4).

**Conclusion:**

In this small pilot study, an LLM demonstrated moderate performance on identifying PWID. The performance would likely limit usability in screening cohorts with real-world prevalence of IDU (1-10%). Future work will seek improved performance by refining the LLM prompt, evaluating other LLMs, and examining additional data (eg, ID consultation notes). Additional validation is needed with larger, distinct datasets. LLMs holds promise to identify hospitalized PWID to improve health outcomes.

**Disclosures:**

All Authors: No reported disclosures

